# Recent Developments in the Design of Non-Biofouling Coatings for Nanoparticles and Surfaces

**DOI:** 10.3390/ijms21031007

**Published:** 2020-02-03

**Authors:** Carlos Sanchez-Cano, Mónica Carril

**Affiliations:** 1 Center for Cooperative Research in Biomaterials (CIC biomaGUNE), Basque Research and Technology Alliance (BRTA), Paseo de Miramon 182, 20014 Donostia San Sebastián, Spain; csanchez@cicbiomagune.es; 2 Instituto Biofisika UPV/EHU, CSIC, Barrio Sarriena s/n, Leioa, E-48940 Bizkaia, Spain; 3 Departamento de Bioquímica y Biología Molecular, UPV/EHU, Barrio Sarriena s/n, Leioa, E-48940 Bizkaia, Spain; 4 Ikerbasque, Basque Foundation for Science, 48013 Bilbao, Spain

**Keywords:** protein corona, anti-fouling, non-biofouling, PEG, zwitterion, fluorine, nanoparticles

## Abstract

Biofouling is a major issue in the field of nanomedicine and consists of the spontaneous and unwanted adsorption of biomolecules on engineered surfaces. In a biological context and referring to nanoparticles (NPs) acting as nanomedicines, the adsorption of biomolecules found in blood (mostly proteins) is known as protein corona. On the one hand, the protein corona, as it covers the NPs’ surface, can be considered the biological identity of engineered NPs, because the corona is what cells will “see” instead of the underlying NPs. As such, the protein corona will influence the fate, integrity, and performance of NPs in vivo. On the other hand, the physicochemical properties of the engineered NPs, such as their size, shape, charge, or hydrophobicity, will influence the identity of the proteins attracted to their surface. In this context, the design of coatings for NPs and surfaces that avoid biofouling is an active field of research. The gold standard in the field is the use of polyethylene glycol (PEG) molecules, although zwitterions have also proved to be efficient in preventing protein adhesion and fluorinated molecules are emerging as coatings with interesting properties. Hence, in this review, we will focus on recent examples of anti-biofouling coatings in three main areas, that is, PEGylated, zwitterionic, and fluorinated coatings.

## 1. Introduction

Biofouling is the spontaneous and unwanted adsorption of biomolecules, cells, or microorganisms on engineered surfaces [1]. It is a consequence of the surface properties, such as the charge, hydrophobicity, or coating grafting density, which are derived from either the bulk material or the surface’s coating. In clinical practice, it has a major role in the design and development of surgical implants because biofouling can produce hemolysis, coagulation problems, immunogenicity, and thrombosis, and it is related to bacterial biofilm formation, leading to infections and implant rejections, among other problems [2]. However, it also reduces the sensitivity in biosensors and affects the travelling speed of ships, due to the attachment of marine organisms to ship hulls.

**Figure 1:**
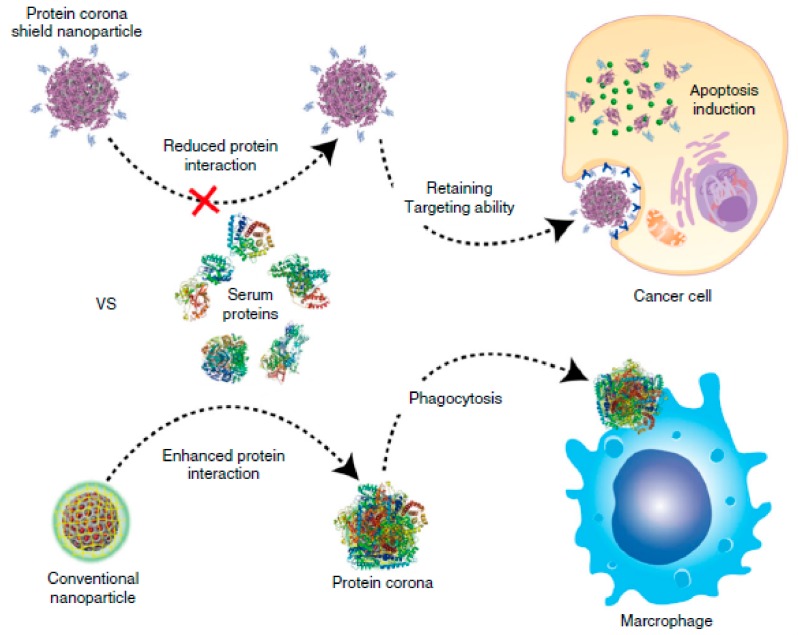
Schematic representation of the influence of the protein corona on the fate and performance of nanomedicines. Reprinted with permission from reference [19]. Copyright (2018) Springer Nature.

In a biological context and referring to nanoparticles (NPs) acting as nanomedicines, the adsorption of biomolecules found in blood (mostly proteins) is known as protein corona [3] and is usually a consequence of the opsonization process that foreign species undergo in the body in order to be recognised as intruders by phagocytic cells (Figure 1). Unlike surfaces, due to their nanometric size, NPs can interact with cells for which new scenarios appear, such as the possibility of being phagocytosed and degraded. Although some studies have suggested that the adhesion of particular proteins could enhance the circulation times of certain NPs by reducing unspecific cellular uptake [4,5], normally, the opsonization process and recognition by the reticulo-endothelial system (RES) substantially shorten the circulation times of nanomedicines in blood, reducing the possibility of them realizing their function, as shown in Figure 1 [6,7]. Hence, understanding the interaction between proteins and NPs or surfaces is a major field of research, for which a plethora of techniques are being developed [8,9,10,11,12].

Protein adhesion is a dynamic process in equilibrium with the surrounding proteins and it changes as the NP environment changes, for instance, from circulating in the blood to cell endocytosis [13] or after crossing biological barriers [14]. Its formation is driven by the affinity coefficient (k_D_) of each protein towards each surface. The proteins with a higher affinity will form the first layer more strongly attached to the NPs’ surface, known as the hard corona. Lower-affinity proteins will mildly interact with the hard corona, conforming what is known as the soft corona [8,15,16,17]. As in the case of surfaces, the interactions involved in protein adhesion on NPs are non-covalent, and proteins mainly interact through electrostatic or hydrophobic interactions. Hence, the physicochemical properties of the engineered NPs, such as their size, shape, charge, hydrophobicity, or curvature, will influence the identity of the proteins attracted to their surface [16]. In this sense, the protein corona, as it covers the NPs’ surface, can be considered as the biological identity of engineered NPs, because the corona is what cells will “see” instead of the underlying NPs. As such, the protein corona will influence the fate, integrity, and performance of NPs in vivo (Figure 1) [7,18].

Although some strategies take advantage of the formation of the protein corona to increase the stability [20] or direct the biodistribution of NPs in vivo [19], in most cases, protein corona formation is considered an undesired process and, hence, approaches for avoiding its formation and increasing the stealth behavior of NPs have been studied. On the contrary, in the fields of coatings for boats, membranes, or surgical materials, biofouling is always a disadvantage and must be avoided. Hence, the design of coatings for NPs and surfaces that avoid biofouling is currently an active field of research. In general, most anti-biofouling coatings seem to adhere to four general principles [21], which are as follows: (i) an overall neutral surface charge; (ii) the presence of hydrogen bond acceptors; (iii) the absence of hydrogen bond donors; and (iv) a high hydrophilicity to promote hydration. The idea behind those principles is to avoid electrostatic or hydrophobic interactions with charged patches or hydrophobic pockets present in proteins and, on the contrary, favor the formation of a layer of water molecules right on top of the engineered surface that will hamper protein adhesion [22]. Nonetheless, there are anti-biofouling surfaces and materials that do not comply to all four of those principles and yet repel the adhesion of biomolecules, such as polyglycerols [23] or polysaccharides [24] that have many hydrogen bond donors or superhydrophobic surfaces [25]. The gold standard in the field is the use of polyethylene glycol (PEG) molecules, although zwitterions have also proved efficient in preventing protein adhesion and fluorinated molecules are emerging as coatings with interesting properties. For those reasons, and although other coatings have been described, such as those based on phospholipids [26] or some saccharides [27,28], in this review, we will focus on recent examples of the design of anti-biofouling coatings in three main areas, that is, PEGylated, zwitterionic, and fluorinated coatings [1].

## 2. PEGylated Coatings

Polyethylene glycol (PEG) is one of the most common antifouling covers used to treat both surfaces and particles. PEG is a hydrophilic polymer which can form dense layers over the surfaces treated. This generates strong steric repulsion with other hydrophilic molecules that allows the adsorption of proteins to be almost completely reduced. Therefore, for a long time, PEG has been considered the gold standard for comparing the efficiency of other antifouling strategies. The capacity of PEG to avoid protein adsorption depends on a number of factors. Among them, the size or length of the PEG chains, the chemical functionalization of their accessible ends, the architecture of the PEG chains (i.e., use of branched PEG structures such as PLL-g-PEGs), and the grafting density of the PEG over the surface covered, are of great importance. A number of experimental articles and reviews have examined the correlation between these factors and the antifouling capacities of PEG layers [29,30,31,32,33,34,35,36]. As such, we will only introduce them briefly when needed, and focus on recent advances and approaches aimed at improving the antifouling properties of PEG covers.

**Figure 2:**
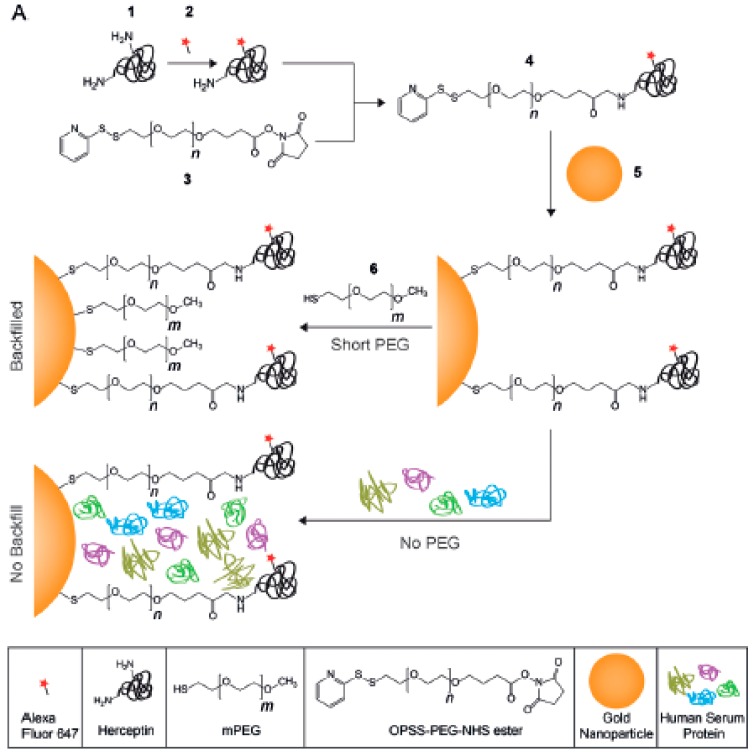
Polyethylene glycol (PEG) chains of different sizes are grafted to Au nanoparticles to generate high-density polymeric shells capable of avoiding the formation of the protein corona. Reprinted with permission from reference [38]. Copyright (2014) Wiley.

The PEGylation of NPs provides an outstanding colloidal and overall stability in biological samples, which is key for their use in biological applications [23,29,30,37]. However, despite their good antifouling capabilities, PEG layers cannot completely avoid the adsorption of proteins. This can negatively affect the properties of PEGylated NPs intended for biological use; for example, by reducing their overall circulation time or hampering their ability to reach the selected target (by covering targeting ligands attached to their surface). An interesting strategy for achieving active targeting is to use PEG chains of different sizes to cover the surface of particles. In this case, short polymeric chains maintain the low adsorption of proteins, while longer PEG chains rise over the protein corona and keep the targeting ligands accessible (Figure 2). It is important to use the right length for short and long PEG, but also to maintain a good ratio between them. For example, Herceptin molecules were used to target 50 nm Au nanoparticles against SKBR3 cells. Herceptin was linked to the particles by 5 kDa PEG chains, which were combined with PEGs with sizes between 1 and 10 kDa (1, 2, 5, and 10 kDa) to form the final polymeric shell. As expected, formation of the protein corona was reduced by all PEG coverages (when compared to non-PEGylated particles), but only particles containing PEGs of 1 and 2 kDa maintained their targeting capabilities [38]. Equally, polymeric Dendron micelles carrying folate ligands on PEGs and non-modified 0.6 kDa PEG chains were capable of avoiding protein corona formation while maintaining targeting capabilities. However, the targeting efficiency of the particles seemed to be inversely related to the percentage of folic acid-2 kDa PEG available [39]. Remarkably, although these changes in targeting capacity might be due to an increase in the interaction between longer PEGs and proteins (as the nature of PEGs changes from hydrophilic to amphiphilic with molecular weight) [40], such an effect is unlikely to happen. When PEG chains are found in a dense brush conformation, such an effect is normally inhibited by steric repulsions.

**Figure 3:**
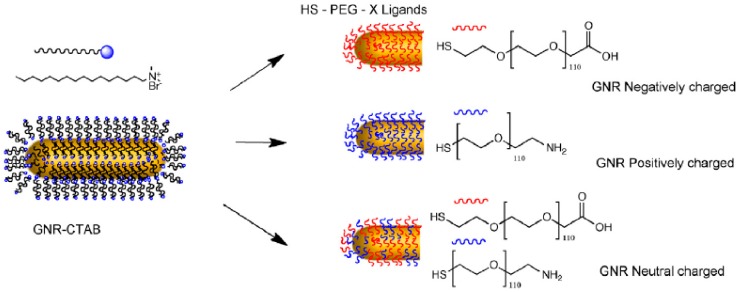
Nanorods covered with 5 kDa PEG chains carrying a -COOH terminal, -NH_2_ terminal, or an equal combination of PEGs carrying both terminal groups show different binding affinities towards Bovine Serum Albumin (BSA). Reprinted with permission from reference [41]. Copyright (2019) Elsevier.

The antifouling properties of particles and surfaces have also been tuned by changing the chemical composition of PEG layers found around them. This includes the introduction of different terminal groups, but also the combination of PEG polymers with other kinds of antifouling covers. The most common chemical modification of the PEG layer of a particle or surface is the use of chains with different charges in their terminal groups. The interactions between such charged PEG layers and proteins would mainly have an electrostatic character. Therefore, although the use of PEG polymers would normally reduce protein binding, the final surface charge will alter the final size and binding strength of the protein corona. For example, in a recent study, gold nanorods were covered with 5 kDa PEG chains carrying a -COOH terminal, -NH_2_ terminal, or equal combination of PEGs carrying both terminal groups (Figure 3). The overall charges for these systems were negative, positive, or neutral (respectively), leading to nanorods with different binding affinities towards Bovine Serum Albumin (BSA), which exhibited the following trend: positively charged PEGylated-nanorods > negatively charged PEGylated-nanorods > neutral charged PEGylated-nanorods [41]. Likewise, a similar effect was observed when CdSe quantum dots (QDs) were coated with 0.6–1 kDa PEG chains containing -NH_2_, -COOH, or -OCH_3_ terminal groups, and their interaction with BSA protein was studied using agarose gel electrophoresis [42]. Again, the binding affinities of NPs with different surface charges towards proteins might be due to changes in hydrophobicity caused by the presence of charged ligands. Nevertheless, the inclusion of both positively and negatively charged ligands in the NPs led to an increase of the hydrophilicity of particles. Therefore, the preferential interaction of proteins with positively charged NPs cannot be directed by changes in hydrophobicity.

Alternatively, the inclusion of small amounts of PEG polymers carrying hydrophobic alkyl chains of different sizes as terminal groups (hexyl-, docecyl-, or octadecyl-PEGs) to 25 nm Au nanoparticles covered by dense PEG layers increased the cellular uptake of particles. Cell internalization was particularly increased for particles with PEGs carrying carbon chain lengths of 12 or longer (dodecyl or octadecyl), but decreased slightly when they were incubated in the presence of 10% fetal bovine serum (FBS). Remarkably, those particles showed a large increase in their hydrodynamic radii in media supplemented with 10% FBS, indicating the formation of a protein corona around the particles. None of these were observed for particles carrying hexyl-PEGs, which behaved quite similarly to normal PEGylated controls and kept their antifouling properties [43].

**Figure 4:**
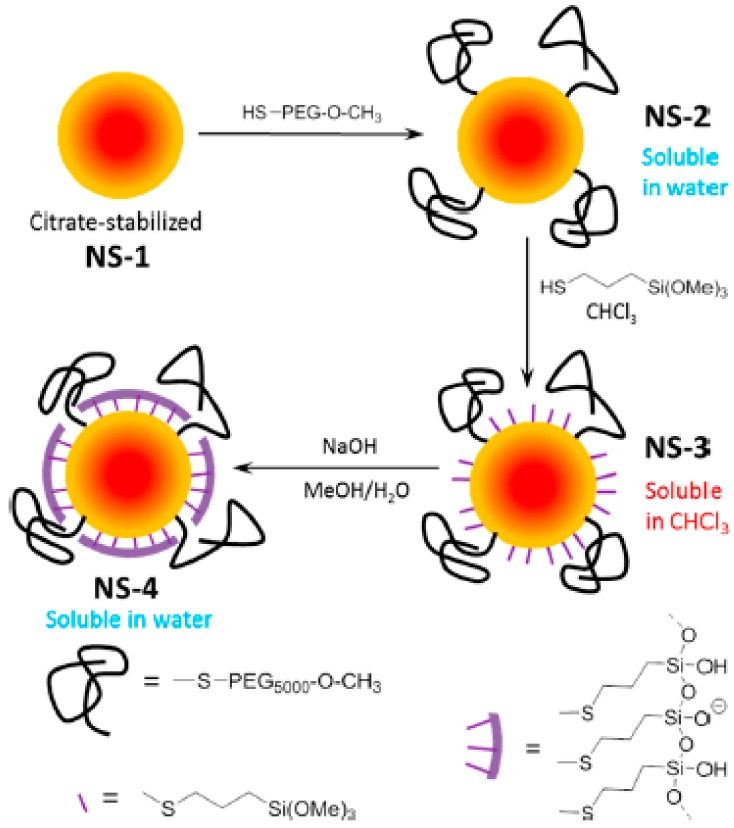
Synthetic protocol of Au nanoparticles carrying a dual PEG-Silica monolayer shell, which provided a high colloidal and chemical stability and improved antifouling properties. Reprinted with permission from reference [44]. Copyright (2019) American Chemical Society.

Finally, PEG polymeric layers have been combined with a single monolayer of silica. Au NPs with a 15 nm diameter were coated with a monolayer of silica by depositing a layer of 3-mercaptopropyltrimethoxysilane monomer on their surface and then crosslinking them using an aqueous basic solution (Figure 4). The synthesis was made possible by pre-stabilizing the particles with a small amount of 5 kDa HS-PEG (0.8 chains/nm^2^). Pre-stabilization with the PEG was needed to allow deposition of the hydrophilic monomer. This permitted particles that combined the properties of PEG (a high aqueous stability, even at a low pH) and silica coatings (stability against cyanides, ligand exchange, and lyophilization, and easy functionalization of the shell) to be obtained. Furthermore, the particles presented much reduced protein coronas when compared with the PEG analogues [44].

Nevertheless, the most important factor governing the adsorption of proteins by PEGylated particles or surfaces is the grafting density of the polymer. The density of PEG in a surface controls the topographical conformation adopted by the polymer chains, which can be described by three parameters: the Flory radius (R_F_) of the grafted PEG, the distance (D) between PEG grafts, and the thickness (L) of the PEG layer. The Flory radius is the radius of the volume occupied by an extended polymer, and is a constant for each type of polymer chain. R_F_ can be calculated using the equation [R_F_ = αN^3/5^] [29], where N is the number of monomers per chain (N) and α is the length of a single monomer (for PEG, α = 0.35 nm). Additionally, the PEG density can be converted to the area that one PEG chain occupies (A), and the distance between PEG graft sites (D) can be calculated using the equation [D = 2(A/π)^1/2^]. Finally, the thickness of the PEG layer can be obtained using different experimental methods (i.e., DLS, TEM, etc.). By knowing the value of the three parameters D, R_F_, and L, the conformation of the PEG chain can be distinguished [45].

**Figure 5:**
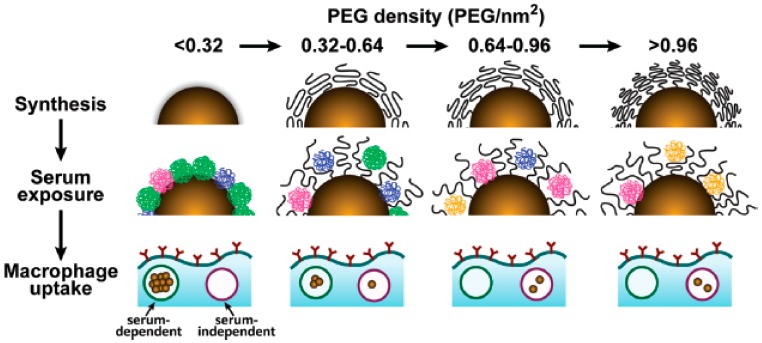
Cartoon showing the relation between the PEG density, protein corona, and macrophage uptake. Reprinted (adapted) with permission from reference [34]. Copyright (2012) American Chemical Society.

If the grafting density of the PEG cover is low, D is larger than R_F_, and PEG chains are present in a condensed conformation (mushroom conformation), where the PEG layer is thin. When the number of PEG chains per surface area increases, the distance between grafted PEGs decreases. This forces the PEG chains into narrower and more extended conformations. When D is smaller than the R_F_, PEG chains adopt a conformation where they are mostly extended (brush conformation), and the PEG layer is much thicker. The interphase between these two conformations is reached when D = R_F_. Furthermore, if the PEG graft density is such that L is larger than 2R_F_, the polymer layers are considered to be in a dense brush conformation. Remarkably, the smaller the particle, the higher the PEG graft density required to reach the brush and dense brush conformation (A is directly related to the curvature of the surface of the particle) [45]. There is a direct correlation between the average volume occupied by each PEG chain and the amount of protein adsorbed by the surface by area unit. This volume depends on the number of PEGs in the surface of the particle, and is directly related to the area A and the curvature of the surface. Therefore, the amount of protein adsorbed by a surface by area unit is inversely related to the particle size and the PEG graft density (Figure 5). As such, achieving a PEG density that led to the brush conformation was vital to reducing the formation of the protein corona [34].

However, even at a high grafting density, PEG covers cannot completely eliminate protein adsorption. For example, serum proteins, specifically albumin, have been shown to adsorb to the dense polymer brush covering superparamagnetic iron oxide nanoparticles (SPION) with cores smaller than 10 nm and PEG polymers grafted at densities ensuring a brush conformation. Only a few of these proteins were found to bind per particle, but they did so with a high affinity (micromolar dissociation constants), which caused almost irreversible adsorption [46]. Similarly, when PEG polymers were covalently attached at the maximum grafting density possible to the surface of polymer-coated nanoparticles, the particles bound to Human Serum Albumin (HSA) with a low affinity, but to fibrinogen (FIB) with a high affinity. Furthermore, changes observed in the hydrodynamic radius confirmed the formation of a protein corona for the PEGylated nanoparticles. However, fluorescence lifetime and quenching measurements indicated that the proteins were embedded within the PEG layer [47]. Finally, recent studies using single molecule fluorescence resonance energy transfer (FRET) analysis showed that increasing the PEG grafting density (using 5 kDa PEG polymers with -OCH_3_ terminal groups) on a surface caused a decrease in the adsorption rate constants of fibronectin, which were embedded in the PEG coating. However, it also resulted in augmentation of the unfolding of proteins adsorbed, and an increase of the retention times of unfolded protein molecules within the polymeric layer [48].

**Figure 6:**
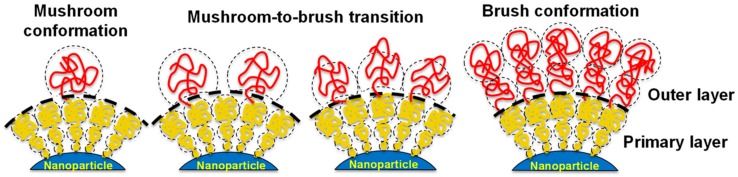
Polymeric nanoparticles carrying a double PEG layer with different graft densities and topographical conformations, which alter the kinetics of the interaction between the particles and proteins. Reprinted with permission from reference [49]. Copyright (2018) American Chemical Society.

This incapacity of simple PEG layers to completely avoid the adsorption of proteins caused the development of new approaches for generating alternative PEG shells with improved antifouling properties. For example, the strategy of using PEGs of different sizes previously used to produce antifouling covers capable of maintaining their active targeting capacities was employed as inspiration to generate particles carrying a double layer of PEG shells. Core-shell polymeric nanoparticles (50 nm) were created using a combination of poly(lactic-co-glycolic acid)-b-poly(ethylene glycol) diblock copolymers (PLGA-PEG: 20 kDa PLGA and 5 kDa PEG), and different percentages of the same block copolymers containing a maleimide-termination. These reactive groups were used to couple 2 kDa HS-PEG-OCH_3_ chains with the surface of the core-shell particles, in order to form a secondary PEG layer on top of the nanoparticle (Figure 6). Measurements of the interaction of these nanoparticles with fetal bovine serum showed that all the PEGylated particles generated adsorbed a similar quantity of proteins, which was smaller than the amount of protein found in the non-PEGylated particles (as previously shown). However, particles with a double layer of PEG bound to proteins with different binding affinities [49].

The lowest binding affinities were achieved when only 20% of the PEG in the first shell was modified with a secondary PEG. This was also the quantity of PEG that showed the longest circulation time. The chain densities in the outer PEG layer were evaluated using fluorescently-labeled 2 kDa HS-PEG-OCH_3_. This allowed the distance between neighboring chains (D) and the Flory radius (R_F_) to be calculated, as described previously [45]. Remarkably, a 20% coverage of the PEGs in the first shell led to a secondary shell that was in the interface between mushroom and brush conformations. In this conformation, the PEG mushrooms were close to each other, but had no substantial lateral interactions. This could be due to the presence of conformational fluctuations of PEG chains that occur at low-density ratios (mushroom to brush), which would ultimately slow down the kinetics of protein adsorption. When the graft density was higher, the PEG chains were confined by their interaction with neighboring polymers, and did not cause the same kinetic effect. Nevertheless, a high grafting density provided strong steric repulsion, needed to achieve low binding of fetal bovine serum. Therefore, the dual-layer design seemed to maximize both effects by combining a dense inner (primary) PEG layer (preventing proteins from being adsorbed by nanoparticles via steric repulsion) with an outer layer of PEG in the mushroom to brush regime (slowed down the adsorption of proteins by nanoparticles by harnessing the conformational fluctuation between topographical conformations of the PEG ligands) [49].

**Figure 7:**
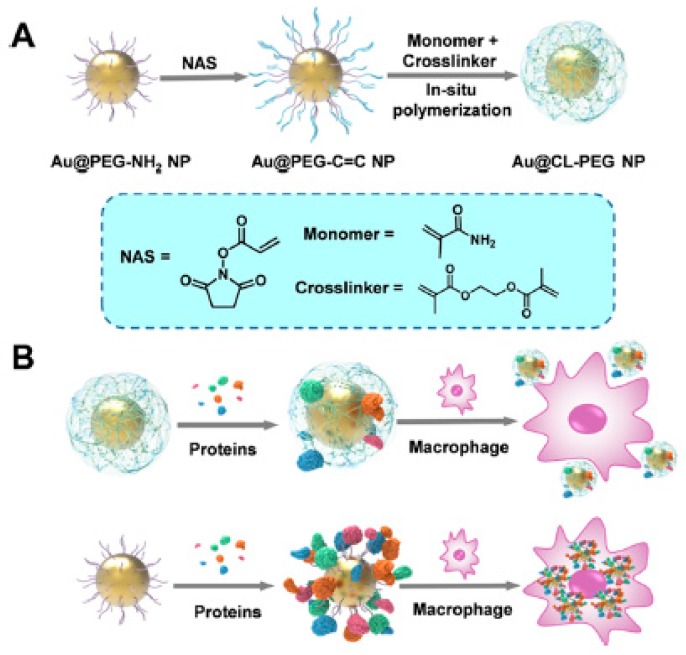
(**A**) Synthetic process leading to the generation of crosslinked PEG shells around Au nanoparticles, and (**B**) representation of the interaction between particles with crosslinked or linear PEG shells and proteins or macrophages. Reprinted with permission from reference [50]. Copyright (2019) American Chemical Society.

Following a different approach, the PEG shell was crosslinked to form a more rigid structure, which led to more inert surfaces. As such, a series of Au nanoparticles carrying a crosslinked PEG shell were developed (Figure 7). The crosslinked PEG shells were formed on the Au nanoparticle cores through in situ polymerization, which led to a higher capability to resist protein corona formation than their linear counterparts without cross-linking. The average quantity of proteins adsorbed per crosslinked nanoparticle was in the range of 2.993 × 10^6^ Da, which was almost half of the quantity of proteins adsorbed by non-crosslinked PEGylated analogue nanoparticles (5.723 × 10^6^, 4.743 × 10^6^, and 5.251 × 10^6^ Da, respectively for Au nanoparticles carrying PEG-NH_2_, PEG-OCH_3_, or PEG-C=C). Furthermore, by crosslinking the PEG shell, the particles obtained a high chemical stability in harsh conditions or biologically relevant media. Remarkably, these particles demonstrated an enhanced stealth effect and colloidal stability under different environments when compared with non-crosslinked analogues [50].

**Figure 8:**
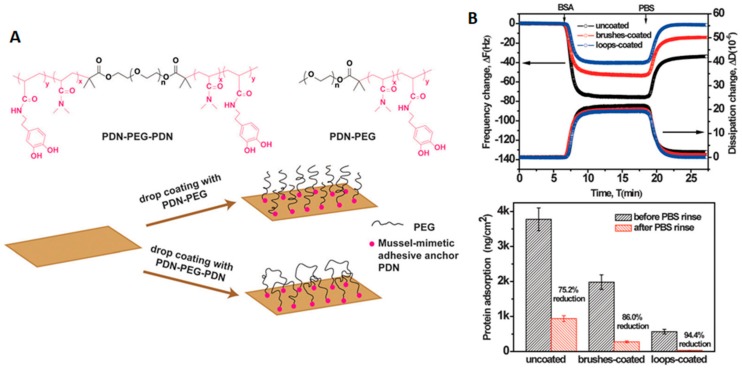
Antifouling loop-forming triblock copolymers. (**A**) structure of loop and linear polymers and representation of their use for coating a surface. (**B**) top: QCM-D traces showing changes in frequency and dissipation upon the absorption of BSA on surfaces uncoated or coated with loop or lineal PEG, and bottom: quantification of protein adsorption on the same surfaces (ng/cm). Reprinted (adapted) with permission from reference [51]. Copyright (2015) Royal Chemical Society.

In addition to the success demonstrated by the crosslinking of the PEG shell, a few groups have recently started to look at the possibility of controlling protein adsorption in surfaces by altering the morphology or topology of the polymers used. For example, instead of normal brushes on the surface of materials, triblock polymers of the type ABA have been used to form polymeric brushes consisting of looped polymers. Normally, these polymers were attached to the surfaces using mussel bio-inspired 3, 4-dihydroxyphenyl-L-alanine (DOPA)-rich polymers on their ends (which can bind to the surfaces that they are grafted to). For example, surfaces treated with loop-forming tri-block copolymers carrying a double-length PEG (Figure 8) showed almost a 10 times reduction in the amount of HSA absorbed (31.7 ± 2.9 ng/cm^2^) when compared with surfaces treated with an analogue diblock copolymer (277.2 ± 32.4 ng/cm^2^). This reduction in the adsorption of HSA was more than 30 times lower than when compared with uncoated surfaces (937.2 ± 82.3 ng/cm^2^). Remarkably, both PEG coatings adopted the brush regime, and the graft density of the loop-forming triblock was half that of the brush-forming diblock, meaning that they had the same end graft density. Therefore, the antifouling differences were mostly due to strong steric hindrance of the neutrally charged polymer loops compared to the brush [51].

**Figure 9:**
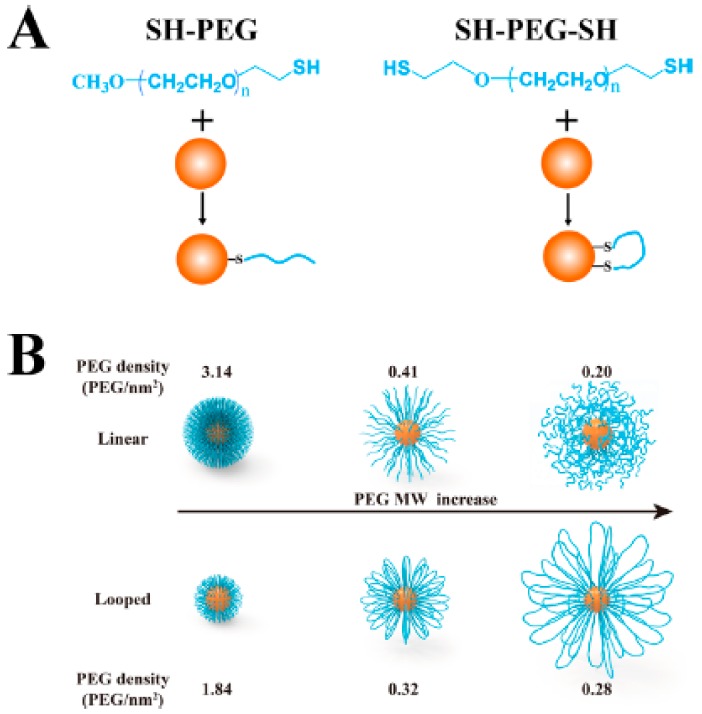
Au nanoparticles carrying linear or loop PEG shells. (**A**) Structure of linear and loop PEG ligands and binding over Au nanoparticle surface. (**B**) Au nanoparticles with linear (top) or loop (bottom) PEGs of different sizes (1, 5, or 10 kDa; from left to right) representing changes in the graft density and hydrodynamic radii of the different polymeric shells. Reprinted with permission from reference [52]. Copyright (2019) American Chemical Society.

Similar results were obtained when linear and looped polymer brush shells based on PEG-SH and SH-PEG-SH chains were formed around Au nanoparticles (Figure 9). In general, looped PEGs on Au nanoparticles were more stable in biological mediums and had better biocompatibility and antifouling properties. Looped polymer shells formed by low molecular weight PEG chains (1–5 kDa) showed smaller hydrodynamic radii and a lower grafting density than the analogue linear polymer shells. Nevertheless, the looped polymer configuration seemed to alter the physicochemical properties of the brush shells when compared with brushes formed by analogue linear polymers. This was observed as loop-forming shells showed the same antifouling properties as the linear PEGs, even at a lower PEG graft density and thickness. Furthermore, when heavier PEG chains were used (10 kDa), loop shells were larger and more densely packed than their linear analogues, and provided better antifouling properties compared with the linear shells [52].

**Figure 10:**
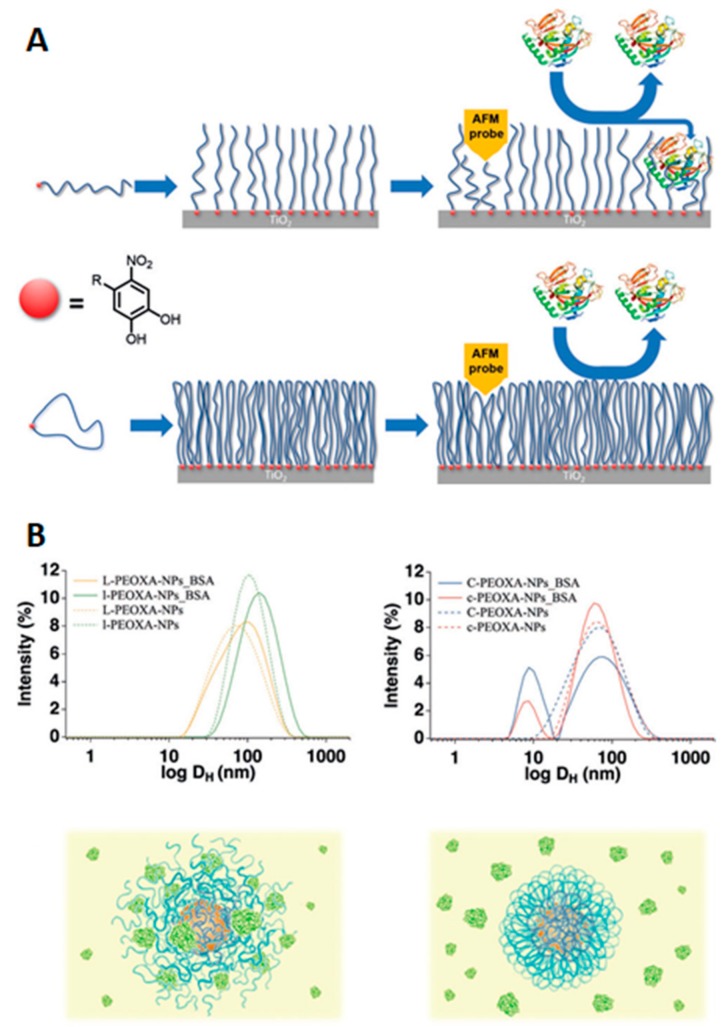
Antifouling cyclic poly-2-ethyl-2-oxazoline (PEOXA) and its effect on protein adsorption. (**A**) surfaces coated with linear (top) or cyclic (bottom) polymers and representation of their interaction with proteins. Reprinted with permission from reference [53]. Copyright (2017) Wiley. (**B**) DLS traces and schematic representation showing the interaction of BSA with nanoparticles coated with linear (**left**) or cyclic (**right**) polymers. Reprinted with permission from reference [57]. Copyright (2017) Wiley.

An equivalent approach to the formation of loops is the use of cyclic peptides for the coating of surfaces and nanoparticles (Figure 10) [53,54,55,56,57]. Again, the cyclic polymer topology alters the physicochemical properties of the brushes. The smaller hydrodynamic radius of ring polymers leads to the formation of denser brushes when compared with linear polymer analogues. This, together with the absence of end-groups (which could interact with proteins), means that coatings prepared from such cyclic polymers outperform their linear counterparts in terms of their antifouling properties. For example, when short and medium cyclic peptides were anchored to titanium oxide surfaces, they showed up to a 50% decrease in the amount of proteins adsorbed (when in the presence of individual proteins or whole human serum) compared to similar brushes from lineal polymers. On the contrary, longer cyclic and linear brushes showed similar behavior, as both of them almost completely avoided the adherence of proteins [56]. Equally, superparamagnetic iron oxide nanoparticles coated with cyclic poly-2-ethyl-2-oxazoline (PEOXA) ligands showed a better colloidal and chemical stability when compared with particles coated by linear analogue polymers. Furthermore, linear polymer coatings were not able to avoid the absorption of BSA by the iron oxide nanoparticles, while the thicker shield provided by cyclic polymer brushes allowed nonspecific interaction between the particles and proteins to be inhibited [57].

## 3. Zwitterions

**Figure 11:**
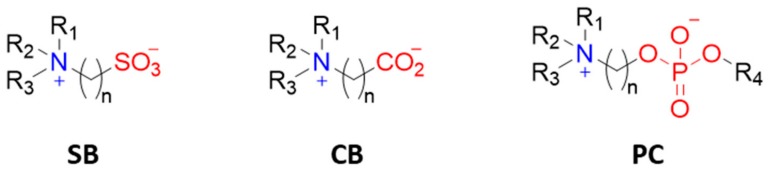
Most commonly used zwitterionic moieties: sulfobetaine (**SB**), carboxybetaine (**CB**), and phophorylcholine (**PC**).

Zwitterions are organic molecules that have both positively and negatively charged moieties close to one another and hence display an overall neutral charge. The positively charged moiety is usually a quaternary ammonium group and the negatively charged moiety can be a carboxylate, sulfonate, or phosphoryl group (Figure 11). Zwitterion ending coatings (ligands or polymers) have been extensively used in anti-biofouling materials [58,59]. The explanation for their great performance as protein repellents is based on their high hydration capability due to coulombic forces between water molecules and the charged moieties in zwitterions. That type of interaction is stronger than hydrogen bonding and, apparently, enough to prevent proteins from binding [22]. In this sense, the selection of functional groups and the number of carbon units between the zwitterionic positive and negative counterparts plays a role in hydration layer formation and hence in protein (non-)adhesion. Amino acids in the right pH range are an example of natural zwitterions, but many more can be obtained synthetically, with sulfobetaine (SB) and carboxybetaine (CB) being the most commonly used frameworks due to their ease of preparation. Phosphorylcholine (PC)-type zwitterions, which resemble cell membranes, are also good anti-biofouling agents, but their preparation is expensive and they are thus less attractive for big surface coatings.

**Figure 12:**
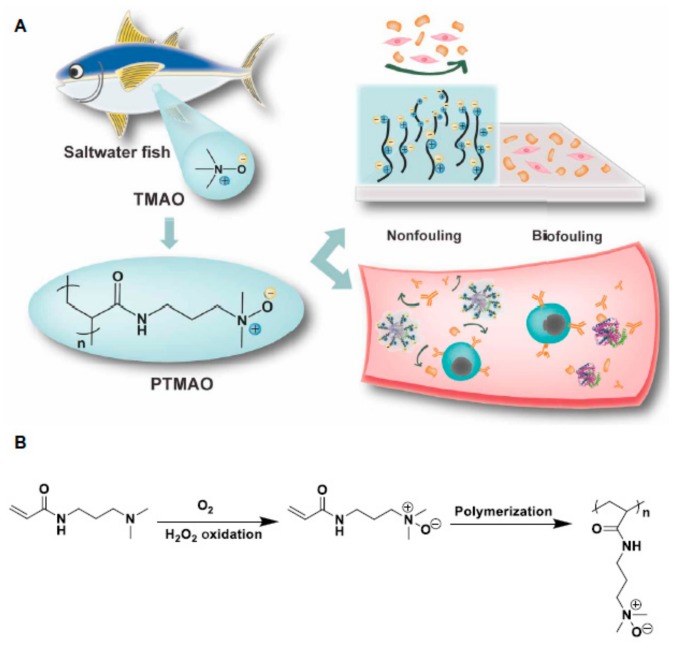
(**A**) Schematic representation of trimethylamine N-oxide (TMAO)-based polymer (PTMAO) anti-biofouling properties when forming hydrogels and (**B**) its synthesis. Reprinted with permission from reference [60]. Copyright (2019) American Association for the Advancement of Science.

However, new zwitterions are being developed. Apparently, as the distance between the oppositely charged moieties decreases, the level of hydration (number of bound water molecules) increases and the non-fouling properties are enhanced. With this principle in mind, Li et al. [60] recently reported the preparation and use of polymers derived from trimethylamine N-oxide (TMAO)—a naturally occurring zwitterion in saltwater fish—in which the charged groups are linked to each other without any spacer between them (Figure 12). A TMAO-based polymer (PTMAO) was prepared as a fourth generation of zwitterionic anti-fouling agents. A hydrogel of PTMAO was prepared and its outstanding non-fouling properties were successfully observed in vitro; firstly upon exposure to a highly concentrated solution of fibrinogen (above physiological concentrations), employing polypropylene as a positive control, and secondly, in undiluted human blood serum. PTMAO displayed 97.6% less fibrinogen adhesion than polypropylene and below 3 ng/cm^3^ serum protein adsorption (anything below 5 ng/cm^3^ is considered non-fouling). It also substantially reduced cell adhesion tested with fibroblasts, which is a major issue in medical implants. Almost non immunogenicity and extended circulation times in vivo were also observed for PTMAO and the hydration as a non-fouling and stealth mechanism was confirmed by molecular dynamics simulations.

In addition to the hydration layer, neutral or zero net charge is proposed as a key parameter for displaying non-fouling properties in surfaces. In this sense, some authors have shown that coatings with positively and negatively charged ligands in a 1:1 ratio also resist protein adhesion [61], even though they are not zwitterionic. Guo et al. [62] studied how the net charge affects the fouling of zwitterionic polymer brushes in relation to the pH. Depending on the charged moieties involved in the zwitterion, the net charge might be pH-dependent, and the pH at which zwitterions will be used as non-fouling coatings must thus be considered. They used polysulfobetaine methacrylate (pSBMA) brushes and observed that the surface ζ–potential was -40 mV, irrespective of the pH, due to the contribution of outer sulfonate groups. They proved that by means of the inclusion of the right amount of cationic moieties in the brushes, they could achieve a zero net charge. Interestingly, when using zwitterions, they observed that the BSA or bacterial adhesion was very low and at similar levels for both only zwitterionic pSBMA brushes with an overall negatively charged surface and those with extra cationic moieties to achieve zero surface charge. Hence, when employing zwitterionic ligands, the hydration layer seems to be more relevant than the actual surface charge. These observations were reported for polymer brushes on planar surfaces. However, in NPs, the inclusion of charged moieties had a deleterious effect on the non-fouling properties. Therefore, other physical parameters related to shape and size (e.g., curvature or roughness) may play a role when studying planar surfaces versus NPs [63].

**Figure 13:**
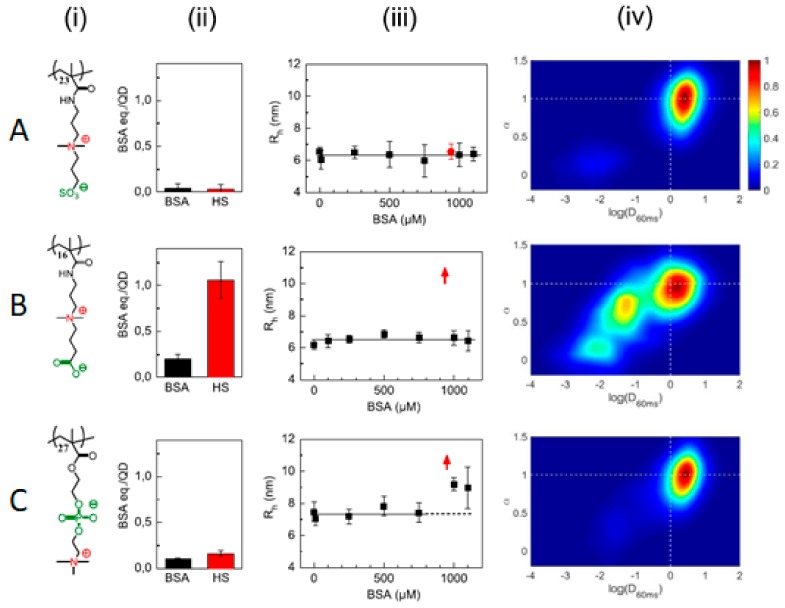
(**A**) Sulfobetaine (SB)-, (**B**) carboxybetaine (CB)-, and (**C**) phosphorycholine (PC)-based polymers (i) and their interactions with BSA and human serum (HS), as probed by a fluorescamine test (ii), size analysis by fluorescence correlation spectroscopy (FCS) (iii), and mobility profiles in the cytosol (iv). Reprinted with permission from reference [58]. Copyright (2019) Elsevier.

The nature of the anionic moieties in zwitterions can also influence their non-fouling capacity in complex environments, such as in human serum or in the cytoplasm of living cells. Sulfobetaine (SB)-, carboxybetaine (CB)-, and phosphorycholine (PC)-functionalized QDs were compared using several techniques, such as the fluorescamine assay or fluorescence correlation spectroscopy (FCS) [58]. For the fluorescamine assay, NP–protein complex isolation is a requirement, for which only strong interactions (hard corona) will be detected. In this sense, SB showed no interaction above the detection limit with both bovine serum albumin (BSA) or human serum (HS), CB showed little interaction with BSA and high interaction with HS, and PC displayed little interaction with both BSA and HS. When the samples were analyzed by FCS to detect the hydrodynamic radius increase due to protein adsorption, it was observed that no changes were detected for SB, which was the coating with the highest non-fouling character, even after a few days of incubation at 37 °C. On the contrary, CB vastly (and irreversibly) aggregated in HS, although it was barely affected by BSA, suggesting that the aggregation was induced by serum proteins other than albumin. On the contrary, PC displayed a size increase both at a high BSA concentration and in HS; however, the small amount of proteins detected by the fluorescamine assay suggested that the size increase was due to weak protein interactions and/or reversible aggregation. When those QDs were internalized in the cytoplasm of HeLa cells, SB- and PC-functionalized ones followed a quasi-Brownian motion profile due to little or no interaction with cytoplasm components, in agreement with their low fouling properties. However, CB-coated QDs displayed slower diffusion coefficients than SB and PC and a mostly non-Brownian motion profile in agreement with the strong interaction with cytoplasm components (Figure 13). According to molecular dynamic studies [64], the number of water molecules in the first hydration layer is highest for SB, followed by PC, and lowest for CB, which may account for the particular non-fouling behavior of each of those zwitterions. Small modifications of the zwitterionic non-fouling SB coating with either ammonium or carboxylate groups led to protein adsorption, which was more pronounced for ammonium moieties. However, neutral biotin inclusion led to no changes in the fouling profile of SB. Hence the hydration layer responsible for the non-fouling capacities of SB seems to be highly correlated with the charge balance between all charged moieties.

**Figure 14:**
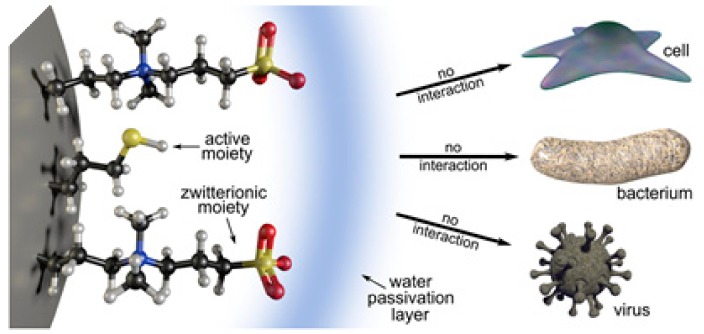
Schematic representation of the shielding effect of the hydration layer due to the presence of zwitterionic moieties. Reprinted with permission from reference [65]. Copyright (2019) Elsevier.

Although zwitterions are effective in avoiding the protein corona and increasing circulation times, they do not have an active targeting capacity towards proteins or membrane receptors. Therefore, the combination of zwitterions with biologically active functionalities for active targeting is highly desirable in the field of nanomedicine, but remains a challenge. It is possible to introduce a secondary functionality, such as amino, carboxyl, or thiol moieties, while maintaining the non-fouling properties of zwitterionic NPs, as long as the zwitterionic groups protrude from the surface by using shorter linker chains for the second functional groups. With such a design, the protective hydration layer typical of zwitterionic materials is maintained, as are the antifouling and stealth features. This was tested with silica NPs and no differences in protein corona formation and the hemolytic capacity were detected between only zwitterionic and doubly-functionalized NPs (with zwitterions and an additional functional group selected from amino, mercapto, or carboxyl groups). Unfortunately, the water molecules attached to the zwitterions actually shielded the secondary functionalities, which were not available for interaction with biomolecules or membranes and were virtually invisible and hence not functional (Figure 14) [65].

However, by balancing the ratio between amino and zwitterionic moieties, it might be possible to maintain both the non-fouling and non-hemolytic properties and the accessibility/reactivity of those amino groups. Bare silica NPs were compared with zwitterionic, amino-functionalized equivalent NPs and amino with either 25% or 75% zwitterionic sulfobetaine ligands. It was observed that as long as there was a percentage of zwitterions on the NPs surface, the anti-hemolytic properties were conserved [66]. On the contrary, both bare and amino functionalized ones produced hemolysis with an increasing NP concentration. Regarding protein adhesion, SDS-PAGE gels showed that 75% zwitterionic NPs behaved as fully zwitterionic ones; that is, no protein corona was detected. On the contrary, 25% zwitterionic and fully amino-functionalized NPs had proteins on their surfaces when exposed to FBS medium. A fluorescamine fluorescent test was used to both quantify the molar amount of amino groups on the surface and probe the accessibility of amino groups for the reaction, despite the shielding effect of the zwitterionic moieties. In all cases, it was possible to perform the test, suggesting that the amino groups were available for a reaction with fluorescamine [66]; however, it must be noted that fluorescamine is a small molecule that can easily diffuse, and the interaction of amino groups with bigger molecules such as peptides or other biomolecules is yet to be demonstrated.

**Figure 15:**
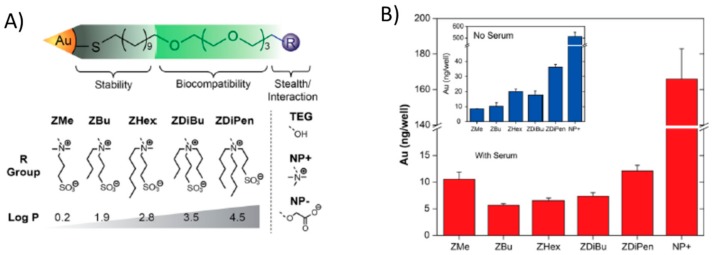
(**A**) Structure of the ligands and zwitterionic/hydrophobic moieties employed. (**B**) Cellular uptake of NPs functionalized with each type of zwitterion in the presence (red) and absence (blue) of serum. Reprinted (adapted) with permission from reference [70]. Copyright (2014) American Chemical Society.

Indeed, the use of zwitterionic coatings, particularly sulfobetaine, can prevent protein corona formation, but as shown above, modifications to the zwitterionic coating can make the non-fouling behavior disappear. Hence, it is difficult to tune the NPs’ surface properties while preventing the protein corona. Apart from the introduction of amino groups, hydrophobicity is an interesting property to tune in NPs due to its role in cellular uptake [67], immune recognition [68], or protein corona formation itself [69]. Modification of the quaternary ammonium moiety of SB with alkyl chains of varied lengths (from one to six carbon atoms) and varied hydrophobicity on 2 nm-sized gold NPs, allowed the hydrophobicity of those NPs without hard corona formation in serum to be tuned [70]. Indeed, either no protein adhesion was detected or reversible adhesion was observed, which disappeared with dilution of the medium. The cellular uptake of both hydrophobic and zwitterionic NPs was not affected by the presence or absence of serum. As the hydrophobicity increased, so did the cellular uptake, regardless of the presence or absence of serum. On the contrary, when only hydrophobic (non-zwitterionic) NPs were used, the formation of a protein corona in serum reduced the cellular uptake relative to the same NPs in the absence of protein corona (serum-free conditions) (Figure 15) [70].

Despite the promising features of zwitterionic coatings, there are few reports on their protein adhesion in complex environments. Ashraf et al. [71] exposed quantum dots (QDs) with zwitterionic, positive, or negative coatings to solutions with an increasing concentration of human serum albumin and measured their size changes due to the protein corona with dual focus fluorescence correlation spectroscopy (dfFCS). Zwitterionic QDs did not suffer from any significant size increase, even at high HSA concentrations, while negatively charged ones did form a monolayer of HSA. Positively-charged QDs aggregated in PBS containing HSA and fluorescence measurements were not possible. The same types of QDs were incubated with HeLa cells at different time points. The maximum uptake was observed for positively charged QDs, followed by negatively charged ones and zwitterionic ones, which were those with the least uptake. The authors suggest that this uptake profile might be related to the presence or absence of a protein corona and will explain the longer circulation times observed for zwitterion-coated NPs.

**Figure 16:**
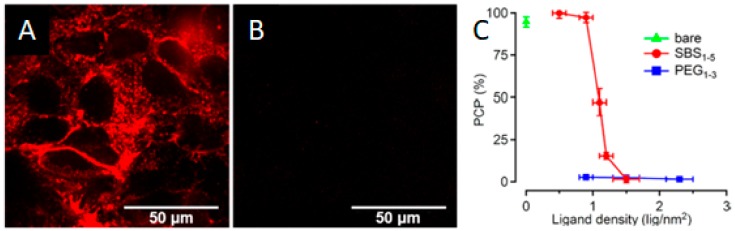
Fluorescence confocal microscopy images of zwitterion-functionalized quantum dots (QDs) (**A**) versus PEGylated QDs (**B**) and the correlation between a positive cell percentage (PCP) and ligand type and density (**C**). Reprinted with permission from reference [72]. Copyright (2019) American Chemical Society.

Indeed, the use of zwitterionic coatings, due to their non-fouling and stealth properties, prevented cellular uptake and hence increased the circulation time of those NPs in vivo. However, Drijvers et al. [72] found that the internalization could be tuned through the ligand density. For 15 nm QDs coated with a silica shell functionalized with sulfobetaines, substantial cellular uptake was detected by both confocal microscopy and flow cytometry when using up to 1 ligand per nm^2^. However, beyond that value, the uptake decreased sharply. On the contrary, the same QDs functionalized with PEG ligands did not internalize at all independently of the ligand density (Figure 16). Although it was not explored, the reason behind this might be that the lower grafting density in sulfobetaines allowed for some interactions with proteins and molecules that favored the endocytosis.

## 4. Fluorinated Hydrophobic Coatings

**Figure 17:**
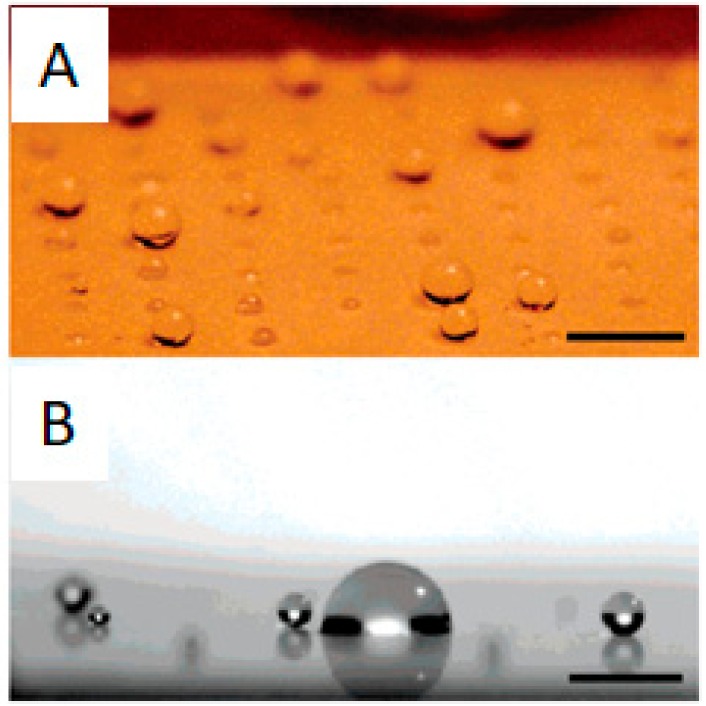
(**A**) Water droplets with different contact angles on a patterned surface with superhydrophobic patches. (**B**) Accumulation of sprayed water droplets on hydrophilic patches on a mixed hydrophilic/superhydrophobic patterned surface. Reprinted with permission from reference [77]. Copyright (2006) American Chemical Society.

Given the existence of hydrophobic patches in proteins, hydrophobicity through hydrophobic interactions plays a major role in protein adhesion. For the case of surfaces, the contact angle is a straightforward way to assess their hydrophobicity and predict their fouling behavior. In this sense, surfaces with contact angles with water of at least 140° and a dynamic sliding angle below 10° are considered superhydrophobic and benefit from the lotus effect (Figure 17). The lotus effect is inspired by lotus leaves in nature, in which the water drops roll and spin on the surfaces (also on the lotus leaf) and remove all dirt and attached microbes or proteins. Angles smaller than 140° provoke sliding of the water drops instead of spinning and this is not that effective in cleaning the surface [73]. A high angle is frequently achieved through a combination of hydrophobic coatings, usually based on perfluorinated alkyl chains, and surface roughness. Indeed, there are numerous reports dealing with superhydrophobic surface preparation methods due to their interesting non-fouling properties [74,75,76].

In fact, more than non-fouling, these surfaces are described as self-cleaning, because biomolecules do adhere, but they are easily removed by the intrinsic nature/features of the surface [1]. This is particularly interesting for marine coatings [78], surgical equipment [73,79], microfluidic devices [25], and the textile industry [80]. Additionally, by combining the superhydrophobicity of the surface coating with interesting photocatalytic surface materials, it is possible to enhance the anti-fouling properties, by not only allowing passive self-cleaning, but also by destroying the organic fouling material under light exposure. For instance, cotton was covered with photoactive TiO_2_, which was subsequently coated with silica modified with an alkyl chain containing eight perfluorinated carbon atoms to confer hydrophobicity to the surface [80]. As the fluorine content on the surface increased, so did the hydrophobicity and the contact angle, but only up to a point, after which the excess of coating presumably altered the roughness of the surface and the contact angle decreased slightly. The self-cleaning possibilities of such surface by the rolling of water droplets were successfully illustrated by using carbon powder as a pollutant, in which the water droplet did not stay on the superhydrophobic surface, but removed the carbon powder by rolling. The photocatalytic cleaning was tested with an organic dye whose stain disappeared under UV light irradiation within 1 h [80].

Fluorinated amphiphilic polymers have been designed as promising anti-fouling coatings for surfaces [81,82]. Controlling the ratio of hydrophilic (2-hydroxyethyl methacrylate) and fluorinated (2-perfluorooctylethyl methacrylate) patches in the copolymer, Zhao et al. [83] were able to avoid protein adhesion for BSA and human FIB. Indeed, by using a percentage of hydrophilic hydroxyl moieties from 4% to 7% and fluorinated moieties ranging from 4% to 14%, protein adhesion was prevented. These values corresponded to surface free energies in the range of 20–30 mN/m, which are known to prevent fouling in marine environments and blood [84].

Therefore, fluorine can have an anti-fouling effect; however, at this stage, it is difficult to translate the lotus effect to fluorinated NPs for in vivo bio-applications as they need to somehow be hydrophilic or water-dispersible. In addition, the curvature of NPs as opposed to flat surfaces can obviously affect the interactions with biomolecules and the performance of fluorinated moieties. Not many NPs with fluorine atoms exposed on the surface have been reported thus far in a biological context and their interactions with proteins have been scarcely studied. For instance, fluorinated quantum dots of a 5 nm core diameter have been used to trap enzymes through hydrophobic interactions [85], for which it could be expected that those fluorinated QDs may form a protein corona. In addition, interfacial tension studies using those same fluorinated QDs showed that they do not prevent protein film formation at the water/oil interface when exposed to bovine serum albumin, apotransferrin, or fibrinogen, although at low protein concentrations, the film formation was slower than in the absence of QDs [86].

**Figure 18:**
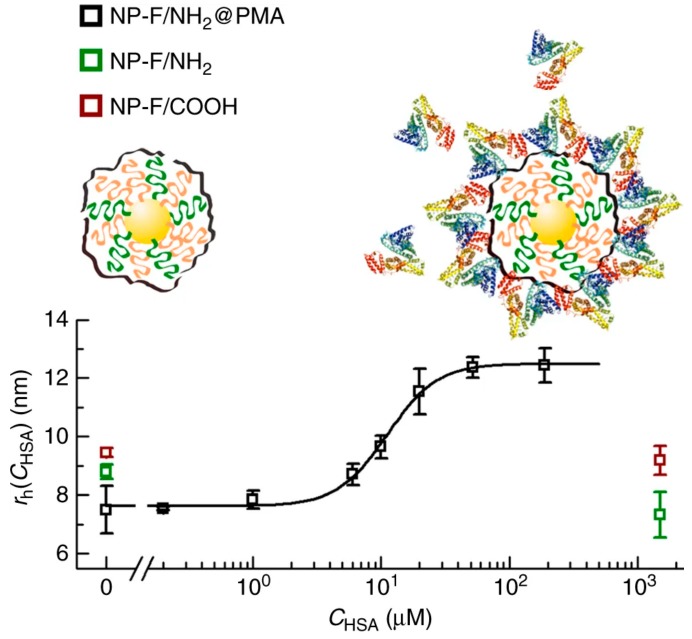
Study of size changes due to Human Serum Albumin (HSA) protein corona formation on fluorinated nanoparticles (NPs) with amino groups (green), carboxyl groups (red), and coated with polycarboxylated polymer (black). Reprinted with permission from reference [9]. Copyright (2017) Springer Nature.

Fluorinated NPs with 3 kDa PEG linkers were used to study protein corona formation through ^19^F-based diffusion nuclear magnetic resonance (NMR) [9]. Changes in the diffusion coefficients (and hence in the size) of several fluorinated gold NPs were monitored by exposing them to single proteins, such as human serum albumin, but also to more complex media, such as blood, plasma, or cells. When those NPs had their fluorine atoms exposed, no size increase was ever detected. NPs either remained mostly unchanged or a size decrease was observed. NPs functionalized with fluorinated ligands and 25–50% carboxylated ligands did not suffer from size changes in the presence of either HSA or FIB; however, their size decreased in human blood and plasma. Fully fluorinated NPs remained unchanged in the presence of HSA, but suffered from a substantial size decrease in the presence of FIB and in the presence of THP-1-cultured cells. Fluorinated and amino (25%)-functionalized NPs suffered from a size reduction in the presence of HSA and FIB. These size decreases detected for all NP types could be due to interactions with proteins on the surface of NPs that induce the compression of soft PEG linkers, leading to size reduction instead of the expected size increase. However, when those fluorinated NPs were coated with a polycarboxylated polymer that avoided fluorine exposition to the outer medium, protein coronas were formed and a size increase was detected in both the presence of single proteins and blood/plasma (Figure 18) [9]. Hence, the fluorine atoms somehow influence the NP–protein interactions.

Surprisingly, even though fluorinated moieties can in principle attract proteins through hydrophobic interactions, they are available to interact and cross several biological barriers precisely benefiting from that hydrophobic character. On the one hand, fluorinated dendrimers were reported to effectively enhance genetic material delivery by means of an increased uptake and fast endosomal escape [87,88]. On the other hand, fluorinated QDs were internalized more than equivalent non-fluorinated QDs, as observed by confocal microscopy, although this might be due to the fluorine-mediated association of QDs, which led to bigger-sized structures [89,90]. In any case, the role of fluorine on NPs in protein corona formation and how this affects interactions with cells is yet to be unveiled.

In summary, the development of anti-fouling coatings is an active field of research for avoiding undesired non-specific protein adhesion to NPs or surfaces. After many years of PEG usage as the standard antifouling agent, we now know how to control its grafting in terms of density and topology to obtain the best results. However, the fact that PEG can be immunogenic has turned the attention to other morphologies (cyclic PEGs) and other antifouling agents, such as zwitterions and fluorinated superhydrophobic surfaces, which have proved to be extremely efficient.

A direct comparison of all coatings is complicated due to the multiple factors implicated, apart from the chemical nature of the coating, such as the underlying material (surface vs. NPs), morphology, length, thickness, or grafting density of the coating. In any case, from the four principles mentioned in the introduction that govern the antifouling capacity of coatings, the hydration layer seems to be the most relevant. For the case of PEG and zwitterionic coatings, the presence of an imperturbable hydration layer is crucial for the antifouling ability of both kinds of coatings. In that sense, zwitterionic coatings are apparently more efficient than PEGylated ones in preserving an intact hydration layer, despite the presence of competing protein interactions, although this seems to be highly dependent on the particular coating design studied. On the contrary, for superhydrophobic materials, the key is the absence of such a hydration layer. The ability of the latter materials to repel water hampers protein–surface interactions and allows water-soluble biomolecules to be washed off.

Despite the plethora of studies reported so far, the field of anti-biofouling coatings, particularly for NPs and nanomedicine, is still somehow in its infancy. The protein adhesion to surfaces and NPs in vivo is a complex, highly dynamic process and many aspects of it are yet to be unraveled before translating these findings to clinical practice. Apart from the complexity of the biofouling process, there are still challenges to be addressed from a coating design point of view, such as the polyfunctionalization of zwitterionic surfaces without altering the non-fouling behavior or the efficient translation of fluorine superhydrophobic properties to NPs.
